# Shared Psychotic Disorder in Old Age: Syndrome of Folie à Deux

**DOI:** 10.1155/2022/8811140

**Published:** 2022-05-02

**Authors:** Q. Schopfer, M. Eshmawey

**Affiliations:** Department of Geriatric Psychiatry, Geneva University Hospitals, Switzerland

## Abstract

**Background:**

Shared psychotic disorder is a relatively rare condition first described by Lasegue and Falret in 1877, who named the syndrome “folie à deux” (FAD). This disorder is characterized by the transference of delusional ideas from one individual to another.

**Methods:**

We report the case of a mother and daughter who exhibited shared delusions of persecution after living in near-total isolation for 10 years. Both patients were hospitalized in two different wards, and the combination of separation, supportive, and pharmacological therapy resulted in remission for the mother and partial remission for the daughter.

**Results:**

Both patients were discharged, and a network was created by the psychiatric hospital which activated social workers for outpatient care. We also planned outpatient psychiatric sessions focused on the patients' specific role in the folie à deux disease, led by two different psychiatrists.

**Conclusion:**

FAD has a long history but it is still an unconventional psychiatric diagnosis. This case report sheds light on the clinical features and how to set proper treatment strategies. While the phenomenon of FAD in old age may not be as rare as previously thought, the literature remains scarce.

## 1. Introduction

Shared psychotic disorder is a relatively rare condition first described by Lasegue and Falret in 1877, who named the syndrome “folie à deux” (FAD). This disorder is characterized by the transference of delusional ideas from one individual to another. The classic presentation involves a primary or an inducer, who suffers from a psychotic disorder, who then influences another person, the secondary or the induced, with a specific delusion. In most cases, two people are involved, but some cases of larger groups, i.e., families have been described and named “folie à famille” [1].

Shared psychotic disorder may result in severe psychotic symptoms and complete social withdrawal. The original syndrome described by Lasegue and Falret was defined under the following circumstances: the primary was more intelligent and exhibited dominant behavior over the secondary; both patients had lived in close association and social isolation, and the delusions were nonbizarre. It is also accepted, per this description, that the primary presents an underlying mental illness, whereas the secondary is considered to be mentally sound before the transference of delusional ideas, and these ideas regress rapidly after physical separation of the two subjects, which is considered to be an important aspect of the treatment [2].

## 2. Case

Mrs. F, a woman in her early seventies, is the mother of Miss G, in her early fifties. Both are immigrants and lived together in the same apartment since the birth of Miss G, with the whole familial structure composed by mother, father, and single daughter. Mrs. F and her daughter were known to be of good social and professional standing in their neighborhood.

In the patients' personal history, both had normal delivery and good psychomotor development, and no psychiatric history is mentioned. Furthermore, no alcohol or substance consumption is reported.

The husband of Mrs. F died in 2008 of cardiac arrest. Miss G revealed that since her father had died, both patients had much difficulty taking care of themselves. It appears as though the death of the husband was a trigger for the development of the psychiatric symptoms. Neither Mrs. F nor Miss G had undergone prior psychiatric treatment.

Between 2008 and 2018, there were no public records of any of the women. They had seemingly disappeared. Contact with neighbors revealed that the patients seldom left their apartment and had very little contact with other people. They started to isolate themselves from their friends and neighbors and slowly cut down all their social and familial ties. This social withdrawal led to a progressive psychological impairment resulting in shared delusions of persecution, specifically that the people in their surroundings represented a potential danger and harbored harmful intentions towards them. Progressively, this belief became a structured delusion where all contacts with the outside world became an important source of threat. From this moment on, Mrs. F and her daughter lived in complete seclusion, spending most of the time at home without any contact with the outside. That is, for 10 years, Mrs. F had not been out of her apartment. During these years, the daughter went out very sporadically, only to provide basic needs.

One day, an electrician was sent to the patients' apartment for repairs, who upon seeing the state of squalor in which they lived, as well as the poor nutritional state of both women, called an ambulance. Both women were brought to the emergency department.

At their mental state examination, both subjects had no insight about their morbid condition. Both patients showed an apparent age discrepancy. They were not oriented to time and presented with incoherent speech due to their delusional thoughts. Mrs. F was in poor physical condition, presenting with obesity and untreated personal care. The daughter was diagnosed with severe protein-energy malnutrition, iron-deficiency anemia, and acute renal failure and had to be admitted to the intermediate care unit to prevent a refeeding syndrome. Psychiatric assessment revealed thought blocking as well as delusional ideas regarding the caregiving staff. After stabilization of the somatic disorders, Miss G was transferred to a psychiatric in-patient unit.

During interviews with both patients present, it became apparent that the mother was the primary. She exerted substantial authority over her daughter, directing her daughter's answers to our questions. It was revealed that during their seclusion, Miss G gave her rations of food to her mother, further confirming that Mrs. F was in fact the primary.

Mrs. F and Miss G were hospitalized in two different wards, in an effort to loosen their delusional link. They also received tranquilizers and antipsychotic medication to improve anxiety subsequent to hospital admission and to treat their delusions, in order to build a therapeutic relationship. The mother's treatment was successful; within a few weeks, she no longer showed delusions of persecution. The daughter also distanced herself from the initial psychotic beliefs without complete remission. Family contact between the mother and her daughter more or less reverted to normal.

At discharge, the daughter was treated with an antidepressant, benzodiazepines, and a second generation antipsychotic. On the other hand, the mother had a better course, showing important improvement with oral therapy with a second generation antipsychotic. A network was created by the psychiatric hospital which activated social workers for outpatient care. We also planned outpatient psychiatric sessions focused on the patients' specific role in the folie à deux disease, led by two different psychiatrists. Mrs. F and Miss G were discharged on two different dates, in order to maintain the remission obtained during their hospital stay.

## 3. Discussion

Folie à deux is considered to be a rare disease, and the literature is almost exclusively composed of case reports. Incidence has been reported to range from 1.7 to 2.6% of psychiatric hospital admissions. However, several authors have since reported that the disorder may be underdiagnosed, or the primary may receive treatment while the psychiatrist may not be aware that other individuals share the patient's delusions [1].

The diagnosis “Shared psychotic disorder” existed in the DSM-III and -IV; however, in the DSM-5, the diagnosis no longer exists on its own but is classified under other specified schizophrenia spectrum [3]. Psychiatrists proposed diagnostic criteria for folie à deux. These criteria are as follows: (a) the content of delusion should be very similar, (b) the persons involved should share and accept each other's delusions, and (c) the persons involved should be closely associated [1, 4, 6]. In recent years, there has been controversy surrounding this nosological entity. Other authors have since also criticized the semiology related to the diagnostic criteria of shared psychotic disorder. In 2006, Arnone et al. conducted a review of the literature from the years 1993 to 2005. This review concluded that the current diagnostic criteria were too restrictive, and that as a result, many instances of the disorder happen outside the spectrum of these criteria [4].

We reported a case of FAD arisen between first degree relatives where the psychotic partners were mother and daughter, respectively, “primary” and “secondary”. In our case, there was a timing course consistent with the “Folie Communiquée” subtype as described by Gralnick [6], where Mrs. F was first affected by persecutory delusions. The daughter began to passively share these beliefs after some time and became subsequently inducted and actively shared the delusions. Regarding the clinical profile of Mrs. F, our diagnosis was late-onset schizophrenia, as there was no evidence of mental illness before the age of 60 [2, 7]. Regarding the daughter, we concluded to a diagnosis of major depression with severe psychotic symptoms. Patel et al. argued that while current diagnostic criteria described schizophrenia as the only diagnosis in the primary, induced delusional disorder may also occur in other mental illnesses such as affective disorders [5].

Regarding the high percentage of parentage, the genetic factor represents a specific diagnostic challenge. It is difficult for the psychiatrist to detect whether the secondary patient suffers from induced delusions or might independently have developed a psychotic disorder due to genetic heritage [3]. Moreover, schizophrenia, a mental disorder in which genetic influence plays an important role, was diagnosed in both primary and secondary in 11 cases reported in the literature. However, the literature did not indicate any estimation of the degree of genetic contribution [6].

The literature shows that the secondary is generally considered mentally sound and exhibits symptoms only after induction by the primary. Some authors have since hypothesized that the secondary often shows a high level of psychiatric morbidity and that exposure to the primary could act as a trigger for the delusions [6]. In our case, it seems that the death of the husband was the trigger that induced the development of symptoms first in the mother. It is also our assessment that the daughter suffered from more severe symptoms than the mother.

In their review, Arnone et al. identified several risk factors for the disorder. Social isolation has of course been described as a major risk factor since the first description of the disease, but other factors have been identified in the secondary, such as cognitive impairment, language difficulties, and life events [4]. It seems in our case that social isolation and life events, specifically the death of the father of Miss G, were the only identifiable risk factors, seeing as the secondary spoke better French than the primary. They also reported that 97.6% of reported cases occurred within the nuclear family, while only 2.4% occurred in the setting of friendships between genetically unrelated individuals [4]. The familial ties in our dyad thus also constitute a risk factor.

The review conducted by Silveira and Seeman in 1995 found that beyond social isolation, comorbid dementia, depression, and mental retardation were common [6]. The majority of shared psychoses were equally distributed among married couples, siblings, and parent-child dyads. It is also specified that in parent-child dyads, the offspring were secondaries in 73.7% of the cases, which could indicate an increased susceptibility in child inductees [6]. This was also found to be true in our case.

However, during the hospital stay, we noticed that the daughter acted as a disturber by reinforcing the delusional ideas of the mother while visiting her. The situation necessitated strict limitation of visits between wards. The separation of the primary and the secondary helped to reduce shared delusions and to diminish anxiety without complete remission. We reported a better improvement in the primary than the secondary. However, the literature indicates that the secondary tends to improve faster [1]. Talamo et al. reported that separation may increase the risk of adverse outcomes; nevertheless, in our patients, we found that separation was beneficial [7].

We found that combined therapy, including supportive and pharmacological therapy, has been effective. As for the discharge program, the psychiatric hospital staff in collaboration with the colleagues of the outpatient service discharged Mrs. F and her daughter on different dates in order to maintain the obtained clinical remission. Both patients were living together when this report was written.

## 4. Conclusion

FAD has a long history but it is still an unconventional psychiatric diagnosis. This case report sheds light on the clinical features and how to set proper treatment strategies. While the phenomenon of FAD in old age may not be as rare as previously thought, the literature remains scarce. More cases should be reported in order to provide new data, which will allow the reevaluation of current semiology and therapeutic principles.
